# Impact of COVID-19 Pandemic Burnout on Cardiovascular Risk in Healthcare Professionals Study Protocol: A Multicenter Exploratory Longitudinal Study

**DOI:** 10.3389/fmed.2020.571057

**Published:** 2020-12-22

**Authors:** Hashel Al Tunaiji, Mai Al Qubaisi, Murat Dalkilinc, Luciana Aparecida Campos, Nnamdi Valbosco Ugwuoke, Eman Alefishat, Lujain Aloum, Ramzy Ross, Wael Almahmeed, Ovidiu Constantin Baltatu

**Affiliations:** ^1^Zayed Military Hospital, Abu Dhabi, United Arab Emirates; ^2^College of Health Sciences, Abu Dhabi University, Abu Dhabi, United Arab Emirates; ^3^Center of Innovation, Technology and Education (CITE), Sao Jose dos Campos Technology Park, Sao Jose dos Campos, Brazil; ^4^Institute of Biomedical Engineering, Anhembi Morumbi University-Laureate International Universities, Sao Jose dos Campos, Brazil; ^5^College of Medicine & Health Sciences, Khalifa University, Abu Dhabi, United Arab Emirates; ^6^Myriad Global Solutions, Abu Dhabi, United Arab Emirates; ^7^Heart & Vascular Institute, Cleveland Clinic Abu Dhabi, Abu Dhabi, United Arab Emirates

**Keywords:** COVID-19, burnout—professional, cardiovascular risk (CV risk), immune dysfunction, healthcare professional

## Abstract

**Introduction:** The coronavirus disease 2019 (COVID-19) pandemic has created new and unpredictable challenges for healthcare systems. Healthcare professionals are heavily affected by this rapidly changing situation, especially frontline healthcare professionals who are directly engaged in the diagnosis, treatment, and care of patients with COVID-19 and may experience psychological burdens. The objective of this study is to explore the evolution of psychosocial, cardiovascular, and immune markers in healthcare professionals with different levels of exposure to the COVID-19 pandemic.

**Methods and Analysis:** This is a STROBE compliant, blended, exploratory study involving online and onsite approaches that use wearable monitoring. A planned random probability sample of residents, staff physicians, nurses, and auxiliary healthcare professionals will be recruited. The study sample will be stratified by exposure to the COVID-19 pandemic. As a first step, recruitment will be conducted online, with e-consent and using e-surveys with Maslach Burnout Inventory, Fuster-BEWAT score, and sociodemographic characteristics. Onsite visits will be planned for the second step where participants will receive a wearable setup that will measure heart rate, actimetry, and sleep quality monitoring, which will be used together with blood sampling for immune biomarkers. Steps 1 and 2 will then be repeated at 2–3 months, and 6 months. Power BI and Tableau will be used for data visualization, while front-end data capture will be used for data collection using specific survey/questionnaires, which will enable data linkage between e-surveys, internet of things wearable devices, and clinical laboratory data.

**Clinical Trial Registration:** ClinicalTrials.gov; Identifier: NCT04422418

## Introduction

The coronavirus disease 2019 (COVID-19) pandemic has created new and unpredictable challenges for healthcare systems. Healthcare professionals are heavily affected by this rapidly changing situation (1). Common challenges faced by all healthcare modalities during the pandemic include limited expert staff availability and the risk of the peri-procedural transmission of SARS-CoV-2 between patients and staff (2). Managing the unpredictable scenario of the COVID-19 pandemic caused an unprecedented challenge for healthcare leadership to promptly act to reorganize and provide the critical resources and information workforce needed to manage this health crisis. Recommendations on precautions, indications, prioritization, and protection are continuously updated for patients and healthcare professionals (1, 2).

The COVID-19 pandemic has caused striking increases in the prevalence of symptoms of depression, anxiety, and burnout in clinicians and other healthcare professionals (3). Physician burnout is already a significant problem in the healthcare industry (4). Working stress and burnout can lead to chronic symptoms of exhaustion and ultimately to an increased risk of cardiovascular disease (5). The total occupational burden has also been related to cardiovascular risk in physicians (6). For instance, medical occupational risk factors such as stress and adverse psychosocial working conditions can increase cardiovascular disease (7). Increased risk of cardiovascular morbidity and mortality have been identified by alterations in cardiac autonomic function that can be assessed by cardiac variability (HRV) (8). Recent findings suggest that the measurement of HRV can be applied in occupational settings to assess burnout (5). According to Lo et al., measuring HRV “not only allows administrators to quickly select the colleagues who need health care but also provides timely and appropriate care, thereby promoting the health of the worker” (5). Cardiovascular risk is not only increased by work-related stress in healthcare, the immune system can also become deficient (9). Immunological biomarkers have been reported to be associated with job-related stress (10). A single-item global job satisfaction measure may be a valid tool to evaluate immune status in healthy white-collar employees (11).

As Dzau, Kirch, and Nasca discuss in a report on preventing this parallel pandemic, “the surge of physical and emotional harm that amounts to a parallel pandemic, […] there has never been a more important time to invest in the clinician workforce. We have a brief window of opportunity to get ahead of two pandemics, the spread of the virus today and the harm to clinician well-being tomorrow. If we fail, we will pay the price for years to come. In the race to respond to the Covid-19 crisis, we must not neglect to care for those who care for us” (12).

## Aims and Objectives of the Study

The purpose of this project is to explore the development of psychosocial, cardiovascular, and immune markers in healthcare professionals with different levels of COVID-19 exposure. The effects of the pandemic work burden on psychological, cardiovascular, and immune markers will be stratified per level of exposure to the COVID-19 pandemic, diagnostic to COVID-19, profession, sex, age, and already existent cardiovascular risk.

The objectives of the study are: (I) to evaluate the level of burnout and cardiovascular health between frontline and second-line healthcare professionals; (II) to identify the effect of healthcare professional category (residents, staff physicians, nurses, and auxiliary staff) and the number of years of experience in relation to the possible associations between burnout and cardiovascular and immune biomarkers; and (III) to explore the association between burnout and cardiovascular and immune biomarkers in frontline and second-line healthcare professionals in multivariable linear regression models, adjusted for relevant confounding variables, including information on socio-demographic, anthropometric, and traditional CVD risk factors.

## Methods

### Ethics

**Figure 1:**
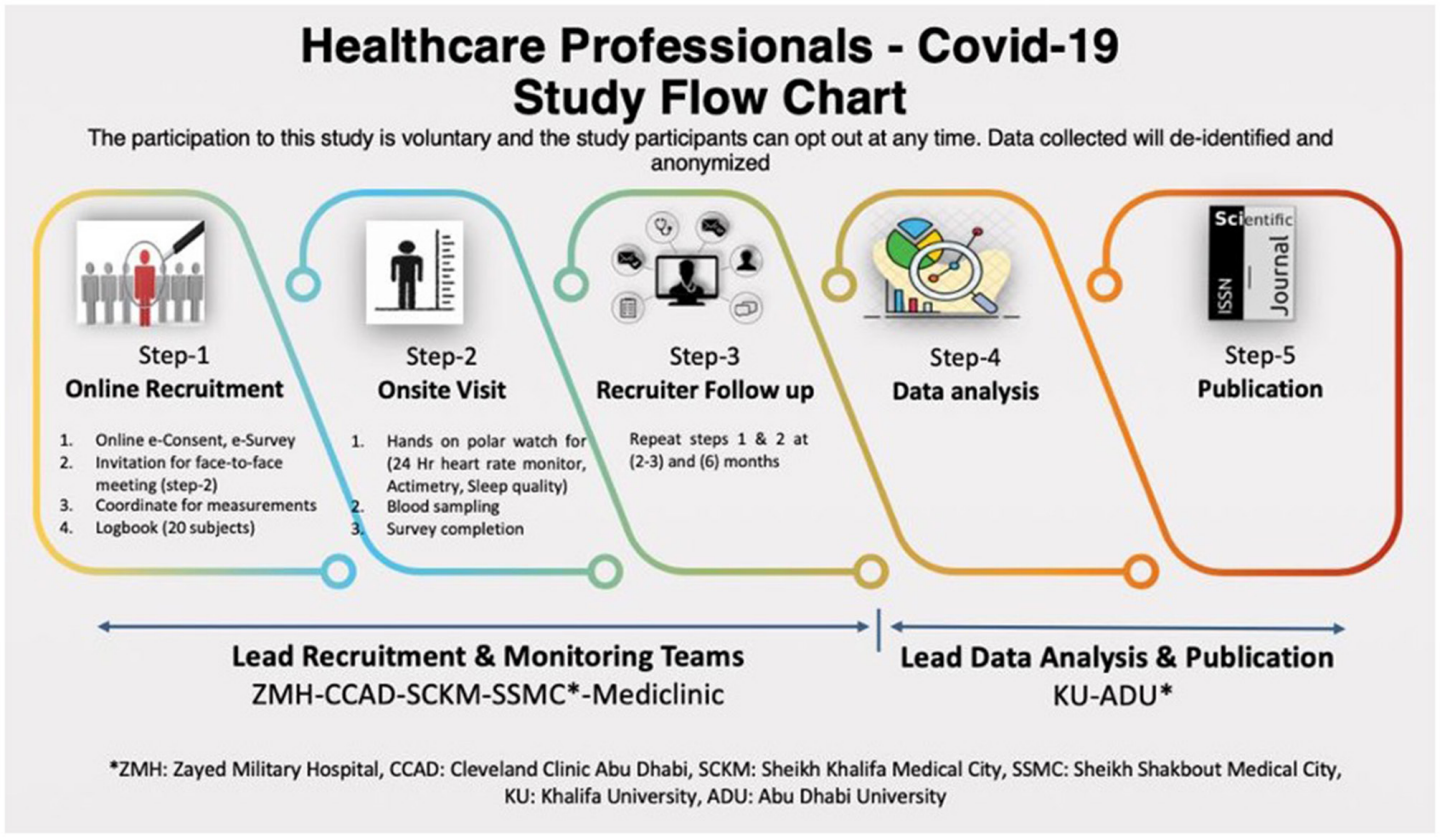
Covid-19 study flow chart.

Electronic informed consent (e-Consent) will be obtained through the initial study recruitment email before the participants complete the online questionnaires (13, 14). Written informed consent will be obtained in the second step of the study (Figure 1) when eligible participants will be verbally informed by trained research personnel about the nature and purpose of the study, and the use of wearable technology for data collection. All participants will be made aware of the fact that they can withdraw from the study at any time without penalty.

### Design

This is an exploratory observational cohort study. The STROBE and The EQUATOR Network were followed in the design of the protocol of this study (15). The study is designed to capture the phenotyping of participants in the areas of psychosocial, cardiovascular, and immune markers profiles, and healthcare usage data linkage.

### Setting

Hospitals in the United Arab Emirates.

### Inclusion and Exclusion Criteria

Participants will be recruited from public hospitals in the UAE. The study has a recruitment goal of 200 residents, staff physicians, nurses and auxiliary healthcare professionals (adult males and females over 18 years) from both inpatient and outpatient medical services will be assessed at baseline, 3 months, and 6 months with a single outcome assessor (Figure 1). A follow-up protocol for participants will be applied to minimize missing data to <20%.

Inclusion criteria are that all residents, staff physicians, nurses, and auxiliary healthcare professionals from both inpatient and outpatient medical services who agree to be part of the study will be provided with heart rate tracking devices to monitor heart rate.

Exclusion criteria will be that participants are not willing to complete the written consent form.

### Recruitment

Potential participants in the study will be recruited through institutional mass email, which will be sent by either a campus office or the researcher (16). The study uses random probability sampling with each population member having a non-zero chance of being selected. In order to increase response rates and facilitate confidentiality, the anonymity of data, and e-consent in participants, it will be necessary to identify recruitment researchers through electronic surveys (17). Since our study is sponsored by academic and governmental agencies, we expect to have good response rates for mass email recruitment (18). Electronic surveys are a widely used method of collecting data in an efficient and timely manner (17), which will facilitate the Maslach Burnout Inventory, the Fuster-BEWAT score, and the accuracy of the sociodemographic characteristics gathered.

### Sample Size

Our study initially explored the ideal sample size based on consideration of the feasibility of recruitment, costs, and logistics. After an initial evaluation, a minimum recommended sample size of 270 healthcare professionals was determined to be valid by Rao Soft® sample size calculator at a 90% power and 5% margin of error. The sample size was 220 when calculated with G^*^Power 3.1.9.6 for Mac OS X G^*^Power 3, an analysis program for the social, behavioral sciences (19), considering an effect size w = 0.3, α err prob = 0.05, power (1-β err prob) = 0.95. To minimize the possibility of attrition bias, we ensured good communication between site principal investigators and participants with personalized invitations (20).

### Data Collection and Assessments

Data will be collected at baseline 3 months and 6 months with a single outcome assessor (Figure 1).

The first step will involve online recruitment using e-consent and an e-survey with Maslach Burnout Inventory, Fuster-BEWAT score, and recording socio-demographic characteristics. During the first stage, we will also plan the onsite visit. The second step will be onsite, and study participants will receive a wearable device for heart rate, actimetry and sleep quality monitoring, and also blood sampling for immune biomarkers. Steps 1 and 2 will be repeated for 2–3 months and 6 months. A follow-up protocol for participants will be applied to minimize missing data to <20%.

The primary outcomes of this study will measure the levels of (I) burnout (*via* survey questionnaire, attached as Supplementary Material 1), through self-reported stress and burnout thoughts, beliefs, emotions, behavior related to Covid-19 using Maslach Burnout Inventory, sociodemographic and anthropometric characteristics (21–23); (II) cardio-vascular risk, through Fuster-BEWAT score (FBS) (24, 25) (included in Supplementary Material 1); through user-friendly mobile technology for short-term HRV deep during breathing test (8); through wearable monitoring technology, measuring 24-h heart rate variability indexes and actimetry suited to report changes in physical activity over time (5, 26), sleep stages length and quality (27); and (III) immune blood biomarkers, recorded through blood samples, which will be collected and analyzed (general health and stress-related markers) by a centralized laboratory (including C-reactive protein, cortisol, blood CBC panel, blood chemistry panel, random blood sugar). Concerning the wearable monitoring technology that the study will use, we evaluated systems that allow data to be exported, with third party compatibility for the scientific data analysis of a continuous heart rate, which also allows for 24/7 activity tracking and sleep tracking, and decided to use smartwatches. Smartwatches with these characteristics are outlined in Supplementary Material 2. We chose the Polar Ignite because this system allows for data to be exported to a third party, and has compatibility for scientific data analysis, and enables continuous 24/7 heart rate capability, which allows the monitoring of activity and sleep tracking. Furthermore, the Polar Team Pro combines actimetry movement data, inertial sensor metrics, and integrated heart rate monitoring in a mobile and easy-to-use wearable tracking system.

### Data Integration and Management

**Figure 2:**
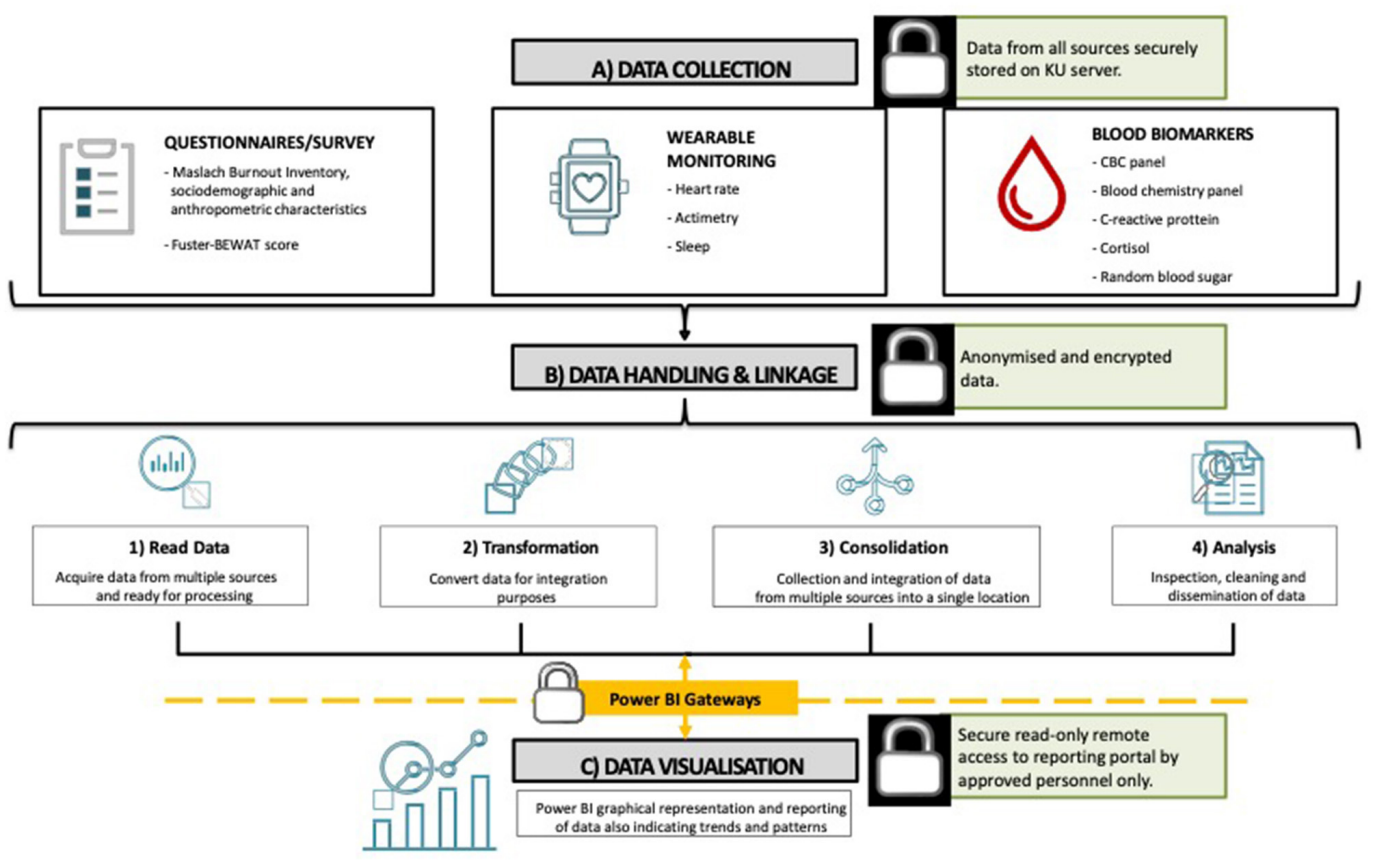
Covid-19 study data integration and management process.

The collection and management of data will consist of 3 distinct steps: a) data collection, b) data handling and linkage, and c) data visualization (Figure 2).

Questionnaire/survey related data will be collected *via* a tool (installed on iPads) that will be built using x-Code, or from existing management and collection tools, and will be designed to appropriately feed specific data into the database for further processing. The data collection tool will include forms to collect all necessary data and import portable document format files (PDF), for scraping, where applicable. All remaining data, including readings from the wearable device and hematology, will be exported from the respective sources in comma-separated values (CSV) format and processed as described in Figure 2. After data handling and linkage, the information will then be processed for visualization purposes (Microsoft Power BI, Washington, USA). The visualization tool will have a dashboard that displays the collected data. The visualization dashboard will enable variables to be filtered and sorted, and observe graphical representations, trends, patterns, and anomalies of the data being analyzed.

All participant information and data generated during this study will be kept confidential in accordance with the HIPAA (Health Insurance Portability and Accountability Act of 1996) (28) on subject privacy and will not be used for any purpose other than conducting the study. Several safeguards will be in place to protect participant information and will be kept anonymous. Participant data will be coded and matched to random numbers to ensure the protection of data. No personal identifiers such as name will be recorded.

### Dissemination of Findings

Data and findings will be presented to healthcare policymakers within Abu Dhabi, to develop preventive strategies that reduce burnout, cardiovascular risk, and immune dysfunction. Data analysis, the release of results, and publication of manuscripts are scheduled to start in early 2021. Findings will be shared in peer-reviewed journals, and at regional, national, and international scientific conferences.

## Discussion

This paper has presented the study protocol for an investigation of burnout, cardiovascular risk, and immune dysfunction in healthcare professionals through a blended online and onsite approach with wearable monitoring for a planned random probability sample of healthcare professionals. Considering that several other similar studies might be undertaken in other countries and that the publication of this study protocol will potentially be used by other institutions in the region, we believe there are potentials for data harmonization and cross-replication.

Strategies to protect clinicians' well-being may involve a blended approach using online and onsite well-being programs, such as heart rate variability biofeedback, internet-based cognitive behavioral therapy, and mindfulness-based resilience training programs (29). Heart rate variability biofeedback has been used to regulate autonomic balance in patients with physical illnesses and mental disorders. A recent study has demonstrated that heart rate variability biofeedback increased autonomic modulation and improved the symptoms of depression and insomnia among patients with major depression disorder (30). A meta-analysis indicated that heart rate variability biofeedback greatly afflicted by anxiety, depression, and anger (31). Smartphone-Delivered Biofeedback Training was effective in increasing resilience while reducing stress and depressive symptoms (32). Heart rate variability biofeedback with resonant frequency breathing on sleep significantly improved the quality of sleep assessed by the Pittsburgh Sleep Quality Index (PSQI) scores (33). Wearable smart device heart rate variability biofeedback shows promise for individualized real-life stress reduction interventions and further and further well-designed trials are warranted (34).

Internet-based cognitive behavioral therapy (I-CBT) can reduce fatigue severity and can be used as the first step in stepped care (35). In an observational study with a pre- and post- treatment study design, internet-based cognitive behavioral therapy (I-CBT) was embedded in stepped care and established as a non-inferior to face-to-face cognitive behavioral therapy (CBT) for chronic fatigue syndrome (CFS) (36). A mindfulness-based resilience training program [the Resilience@Work (RAW) Mindfulness Program] delivered *via* the internet effectively enhanced resilience among a group of high-risk workers in a recent cluster randomized controlled trial (37).

One potential limitation of the present study is that the sample size was not selected to power a specific primary hypothesis and therefore should be considered as exploratory. As on-site visits to hospitals are planned for the second stage of the study protocol, difficulties may arise due to the regulations imposed by the pandemic. Another potential challenge could be due to the fact that the participants in the study may have reservations about the wearable devices, including worries about geophysical tracking data. In order to prevent such difficulties, recruiters will be specially trained to follow the requirements enforced by the pandemic and to provide the participants with sufficient clarifications regarding the study and the equipment used.

Considering the necessary physical and social distancing during a pandemic, internet- and smartphone- technology therapies or preventive measures can be used as an effective intervention or prevention in people with resilience or occupational stress.

The internet could be exploited for telemedicine and restoring daily routines, online platforms may be used to monitor the toll of the pandemic on mental and cardiovascular health (38).

## Patient and Public Involvement

This is an urgent occupational health research study in response to an Occupational Health Emergency and safety of healthcare professionals of International Concern. Study participants, the healthcare professionals were not involved in the design, conduct, or reporting of this rapid response research.

## Ethics Statement

Ethical approval was obtained from the Institutional Review Board of Khalifa University (protocol # CPRA-2020-034), Abu Dhabi COVID19 Research IRB Committee of the Department of Health- Abu Dhabi (DOH/NCVDC/2020/1052), and the Emirates IRB for COVID Research Committee (DOH/CVDC/2020/1246).

## Author Contributions

HA, OB, MA, MD, and LC developed the original concept of the study for the original grant application. OB, HA, and WA prepared the drafts of the study protocol manuscript and compiled feedback and changes from other authors. HA prepared the data model and together with WA are providing leadership for the Investigators Team. HA and OB (Principal Investigators), WA (study chair, healthcare leadership management), MA (project lead, electronic questionnaires, and onsite management), MD (project lead, cardiovascular, and physical activity), LC (project lead, cardiovascular biomarkers, and biostatistics), RR and NU (project leads, digital data management, and linkage) are study co-investigators. EA and LA (data analyses leads) and were all involved in writing the original grant application and IRB approval. All authors have carefully read, contributed to, and approved the final version of the study protocol manuscript.

## Conflict of Interest

The authors declare that the research was conducted in the absence of any commercial or financial relationships that could be construed as a potential conflict of interest.

## Abbreviations

COVID-19: disease caused by a new strain of coronavirus
CO: corona
VI: virus
D: disease
HRV: heart rate variability.
