# Supplementary material for: Impact of COVID-19 Pandemic Burnout on Cardiovascular Risk in Healthcare Professionals Study Protocol: A Multicenter Exploratory Longitudinal Study
Source: Front Med (Lausanne). 2020 Dec 22;7:571057. doi: 10.3389/fmed.2020.571057 (PMC7783289; doi:10.3389/fmed.2020.571057)
Supplement: Supplementary file 1 [file Table_1.docx]

| Multi-Center Prospective Cohort Study: Impact of Burnout on Cardiovascular Health among Frontline Healthcare Professionals During Covid-19 Pandemic in Abu Dhabi Emirate | |
| --- | --- |
| Dear Participant,  Thank you for taking part in this study to help improve actions taken in response to the novel coronavirus pandemic (Covid-19) and to inform the response to similar future outbreaks.  This study will involve answering a 10-15 minutes survey which will be asking you questions relating to the possible effects of coronavirus pandemics on stress, burnout and cardiovascular risk.  Please do not start until you will have enough time to complete it in one go. Please close other programmes (e.g. chat or e-mail) to avoid distractions.  This study is conducted by Dr. Ovidiu C. Baltatu and Dr. Hashel Al Tunaiji and the data is collected by Khalifa University.  By taking part, you are agreeing that you have read and understood the information about the study below. Please ensure you have read and understood this information before continuing.  **What is this project about, and do I have to take part?**  The coronavirus disease 2019 (COVID-19) pandemic has created new and unpredictable challenges for healthcare systems. Healthcare professionals are heavily affected by this rapidly changing situation. They may experience psychological burden, especially health care professionals directly engaged in the diagnosis, treatment, and care for patients with COVID-19. The objective of this study is to investigate the evolution of psychosocial, cardiovascular and immune markers in healthcare professionals with different levels of exposure to the COVID-19 pandemic.  **Study Procedures and Outcomes**  The study outcomes will be collected in 2 steps, online and onsite, that will be repeated at 2-3 months, 6 months.  In the first step, recruitment will be conducted online, with e-consent and e-survey with Maslach Burnout Inventory, Fuster-BEWAT score and sociodemographic characteristics, during this, an onsite visit will be planned. In order to contact you for the onsite visit, you will be asked to provide your moble number and email, which will be not shared with any other organisations or agencies.  In the second step, will be an onsite visit to investigate the heart rate variability, physical activity and screen time and blood sampling:   1. The heart rate variability will be at your choice: the short-term (up to 5 minutes) test with an app installed in the smartphone, or long-term (at least for 24 hours) with a smartwatch Polar that will be setup to monitor 24-hour heart rate without GPS recordings to prevent your geolocation. The Polar watch will be returned at the end of the study. 2. Physical activity and screen time will be collected with your agreement manually from your smartphone: Physical Activity from: Health Data of iPhone or Google Fit of Android; Screen Time: Week Average from iPhone and Android). 3. Blood sampling will be for measurements of C-reactive protein, cortisol, complete blood count panel, lipid profile, urea, creatinine, alkaline phosphatase, and possibly other specific immunoglobulins and interleukins.   Optional, you may ask for a pulmonary function test (PFT: lung volume, capacity, rates of flow, and gas exchange).  A description of this clinical trial is available on <http://www.ClinicalTrials.gov> (Identifier: NCT04422418). This Web site will not include information that can identify you. At most, the Web site will include a summary of the results. You can search this Web site at any time.  **What are the benefits and risks of taking part?**  You may benefit from taking part in the survey by being motivated to look up information about the coronavirus pandemic. We will provide you with good resources at the end of the study. There are no foreseeable risks for you when taking part in the survey other than time spent on the survey and potential discomfort. Should you feel uncomfortable and want to leave the study you are free to do so without any consequences. In case the data collected will suggest you are at increased risk, you will be notified.  **Confidentiality of Research Information**  You will be asked questions about yourself, your own fears and worries relating to the coronavirus pandemic.  All patient information, data and clinical records generated during this study will be kept confidential in accordance with the Health Insurance Portability and Accountability Act (HIPAA) on subject privacy and will not be used for any purpose other than conducting the study.  Several safeguards will be in place to prevent disclosure of patient identifiable information and all personal identifiers will be kept anonymous. Also, all identifiers will be kept in password-protected files on Khalifa University supported computer. Participants identifying information will be coded and matched to specific numbers to ensure protection of personal information. All other sensitive data will be password protected and stored on Zayed Military Hospital drive.  **Concerns**  If you are concerned about this study, or how your data is being processed, or if you would like to contact us about your rights, please get in touch with Dr. Ovidiu C. Baltatu (Khalifa University, [Ovidiu.baltatu@ku.ac.ae](mailto:Ovidiu.baltatu@ku.ac.ae)) or Dr. Hashel Al Tunaiji (Zayed Military Hospital, [dr.hashel@gmail.com](mailto:dr.hashel@gmail.com)).  **Consent**  I understand that:   - My participation is completely voluntary. - All my answers will be used for scientific research to improve actions taken in response to the coronavirus   pandemic and to inform the response to similar future outbreaks.   - My data will be stored securely, however, no personal data will be stored, and my answer will be completely   anonymous.   - My data gathered in this study will be shared with relevant researchers and government agencies. - Because I am submitting anonymous data, it will not be possible to withdraw my answers after they have been   submitted.  Please note that you can stop the survey at any time. This will not entail any penalty, and it will not affect the services (health care services or others) that you receive.  By ticking the box, you are agreeing that you are at least 18 years old, that you have read the information about the study, and that you voluntarily agree to take part in it.  [*] I agree to participate in this study. | |
| **Sociodemographic history questionnaire** | |
| **Section 1: Identification** | |
| Definition of frontline line healthcare provider: a physician, nurse or allied health care professional who are in close contact with Covid-19 patients during the pandemic | |
| Based on the above definition, do you consider yourself a frontline care provider? | - Yes - No |
| Are you, or have you been, infected with the novel coronavirus? | - Yes, tested and the result was positive - Yes, suspected but not confirmed by a test - No, tested and the result was negative - No - Don't know |
| Do you know people in your immediate social environment who are or have been infected with the novel coronavirus? | - Yes, confirmed - Yes, suspected but not confirmed by a test - No, tested and the result was negative - No - Don't know |
| Age group | - < 25 years - 25-30 years - 30-35 years - 35-40 years - 40-45 years - 45-50 years - 50-55 years - 55-60 years - > 65 years |
| Gender | - Male - Female |
| Level of education | - High school or less - Diploma - Bachelor - Master - PhD - GP - Specialist - Consultant |
| Years of experience | - 1-5 years - 5-10 years - 10-15 years - 15-20 years - > 20 years |
| Religion | - Islam - Christianity - Hinduism - Buddhism - Judaism - Irreligion - Other (specify) |
| Marital status | - Married - Single - Divorced - Widow - Other (specify) |
| Emirate of residence | - Abu Dhabi - Dubai - Sharjah - Ajman - Ras Al Khaimah - Fujairah - Umm Al Quwain |
| Housing | - Living alone - Living with family - Living with a room or house mates |
| Ethnic origin | - Arab - Asian - African - Caucasian - Black - Mixed ethnic - Other (specify) |
| Speciality | - Non-surgical speciality   - Family medicine   - Emergency medicine   - Intensive Care Unit (ICU)   - Internal medicine   - Radiologist   - Medical Laboratory   - Others (specify) - Surgical speciality   - General surgery   - Subspecialty   - Others (specify) - Nurses   - Emergency medicine   - Intensive Care Unit (ICU)   - Inpateint wards - Allied health   - Pharmacist   - Physiotherapist   - Microbiology lab technician   - Radiology technician   - Pathology technician   - Others (specify) |
| Weekly work hours | - 30-40 hours - 40-50 hours - 50-60 hours - 60-70 hours - > 70 hours |
| Years in shift work | - 0 - 1-5 years - 5-10 years - 10-15 years - > 15 years |

| **Fuster BEWAT Score (FBS)** | | | | | | | | | |
| --- | --- | --- | --- | --- | --- | --- | --- | --- | --- |
| Fuster-BEWAT score (FBS), is a validated health metric consisting of five modifiable risk factors ([B] blood pressure, [E] exercise, [W] weight, [A] alimentation, [T] tobacco), for promoting optimal cardiovascular health | | | | | | | | | |
| Score | | Criteria description screening | | | | | | | |
| **B** | 0 | SBP ≥ 140 and/or DBP ≥ 90 mmHg | | | | | | | |
|  | 1 | SBP: 130-139 mmHg and/or DBP: 85-89 mmHg | | | | | | | |
|  | 2 | SBP: 120-129 mmHg and/or DBP: 80-84 mmHg | | | | | | | |
|  | 3 | SBP ≤ 120 mmHg and/or DBP: ≤ 80 mmHg | | | | | | | |
| **E** | 0 | < 10 min/week moderate to vigorous activity | | | | | | | |
|  | 1 | < 75 min/week moderate to vigorous activity | | | | | | | |
|  | 2 | 75-150 min/week moderate to vigorous activity | | | | | | | |
|  | 3 | > 150 min/week moderate to vigorous activity | | | | | | | |
| **W** | 0 | >30 kg / m^2^ | | | | | | | |
|  | 1 | 25-29.9 kg / m^2^ | | | | | | | |
|  | 3 | <25 kg / m^2^ | | | | | | | |
| **A** | 0 | 1 fruit/vegetable servings daily | | | | | | | |
|  | 1 | 1-2 fruit/vegetable servings daily | | | | | | | |
|  | 2 | 3-4 fruit/vegetable servings daily | | | | | | | |
|  | 3 | >4 fruit/vegetable servings daily | | | | | | | |
| **T** | 0 | >1 pack/day | | | | | | | |
|  | 1 | <1 pack/day | | | | | | | |
|  | 3 | Non-smoke | | | | | | | |
| **Maslach Burnout Inventory (MBI)** | | | | | | | | | |
| The Maslach Burnout Inverntory (MBI) captures three dimensions of burnout: emotional exhaustion (EE), depersonalization (DP), and personal accomplishment (PA). The questionnaire measures 22 various aspects of professional burnout. Each item will be measured with a five-point Likert scale (1 = never burnout to 6 = everyday burnout) understanding). | | | | | | | | | |
| Question | | | Never | A few times per year | Once a month | A few times per month | Once a week | A few times per week | Every day |
| **Emotional Exhaustion (EE)** | | | 0 | 1 | 2 | 3 | 4 | 5 | 6 |
| I feel emotionally drained from work | | |  |  |  |  |  |  |  |
| I feel used up at the end of the workday | | |  |  |  |  |  |  |  |
| I feel fatigued when get up in the morning | | |  |  |  |  |  |  |  |
| I feel like at the end of the rope | | |  |  |  |  |  |  |  |
| I feel burned out from work | | |  |  |  |  |  |  |  |
| I feel frustrated by job | | |  |  |  |  |  |  |  |
| I feel working too hard on the job | | |  |  |  |  |  |  |  |
| Working with people puts too much stress | | |  |  |  |  |  |  |  |
| Working with patients is a strain | | |  |  |  |  |  |  |  |
| **Personal Accomplishment (PA)** | | | 0 | 1 | 2 | 3 | 4 | 5 | 6 |
| I can easily understand patients’ feelings | | |  |  |  |  |  |  |  |
| I can deal effectively with the patients’ problems | | |  |  |  |  |  |  |  |
| I feel positively influencing people’s lives | | |  |  |  |  |  |  |  |
| I feel very energetic | | |  |  |  |  |  |  |  |
| I can easily create a relaxed atmosphere | | |  |  |  |  |  |  |  |
| I feel exhilarated after working with patients | | |  |  |  |  |  |  |  |
| I have accomplished worthwhile things in job | | |  |  |  |  |  |  |  |
| I deal with emotional problems calmly | | |  |  |  |  |  |  |  |
| **Depersonalization (DP)** | | | 0 | 1 | 2 | 3 | 4 | 5 | 6 |
| I become more callous toward people | | |  |  |  |  |  |  |  |
| I treat patients as impersonal “objects” | | |  |  |  |  |  |  |  |
| I am worried that job is hardening me emotionally | | |  |  |  |  |  |  |  |
| I don’t really care what happens to patients | | |  |  |  |  |  |  |  |
| I feel patients blame for their problems | | |  |  |  |  |  |  |  |
| **Semi-structured Face-to-Face Interview** | | | | | | | | | |
| The respondents will be given the option of participating in a semi-structured face-to-face interview to elicit more information on their stress-related issues during covid-19 pandemic with emphasis on promoting healthcare professionals wellness during covid-19 pandemic. Depending on the number, the sample will be purposively selected representing professional groups and interviews continued to saturation | | | | | | | | | |
| The stress-related questions for semi-structured interview would be as follows: | | | | | | | | | |
| **Emotion regulations** | | | | | | | | | |
| 1. Common fear of acquiring COVID-19; 2. Anxiety about infecting family; 3. Anxiety of being infected during commuting; | | | | | | | | | |
| **Knowledge** | | | | | | | | | |
| 1. Overwhelming information overload 2. Lack of knowledge about infectiosity and virulence; 3. lack of knowledge about prevention and protection; | | | | | | | | | |
| **Behavioural regulations** | | | | | | | | | |
| 1. Feeling of being protected by national and local government; 2. Feeling of being protected by hospital (the protection include taking all reasonable precautions to prevent illness, providing for the care of those who do become ill, reducing malpractice threats for those working in high-risk emergency situations and providing reliable compensation for the families of those who die while fulfilling this duty and attenuating the duty of hospital workers not to become a patient him or herself and so on); | | | | | | | | | |
| **Beliefs of cnsequences** | | | | | | | | | |
| 1. Burden of increase quantity of work; (The workload is markedly escalating) 2. Burden of change of quality of work; 3. Physical exhaustion; 4. Mental exhaustion; 5. Insomnia; 6. Elevated mood; 7. Feeling of being avoided by others; 8. Feeling of being isolated; 9. Feeling of having no choice but to work due to obligation; 10. Burden of child care including lack of nursery. | | | | | | | | | |
| Thank you very much!  Your participation provides valuable insights for all of us to react appropriately in the current novel coronavirus situation and to reach healthcare professionals with useful information in a timely manner.  If you have any questions, please contact Dr. Hashel (email) or Dr Ovidiu (email)  If you have changed your opinion and would like to withdraw your consent to use your data, please click on “Withdraw my consent”.  [*] Withdraw my consent | | | | | | | | | |
