# Supplementary material for: Impact of COVID-19 Pandemic Burnout on Cardiovascular Risk in Healthcare Professionals Study Protocol: A Multicenter Exploratory Longitudinal Study
Source: Front Med (Lausanne). 2020 Dec 22;7:571057. doi: 10.3389/fmed.2020.571057 (PMC7783289; doi:10.3389/fmed.2020.571057)
Supplement: Supplementary file 2 [file Table_2.docx]

Supplement 2. Wrist 24/7 heart rate monitoring watches

|  | Brand | Model | 24/7 wrist HR tracking | Open system/export | HR zones | Battery Watch time | Battery Training time | GPS |
| --- | --- | --- | --- | --- | --- | --- | --- | --- |
|  | Polar | Ignite | YES | YES (via Flow) | YES | 5 days | 17hrs | YES |
|  | Polar | Grit X | YES | YES (via Flow) | YES | 7 days | 40hrs | YES |
|  | Polar | M Series 200/430/600 | YES | YES (via Flow) | YES | 6 days | 1-8hrs | YES |
|  | Polar | A370 | YES | YES (via Flow) | YES | 4 days | 1hr | YES (via phone) |
|  | Polar | Vantage M/V | YES | YES (via Flow) | YES | 5-7 days | 30-40hrs | YES |
|  | Garmin | Vivo active 4 | YES | YES | YES | 7 days | 13hrs | YES |
|  | Garmin | Forerunner series 45/245/645/945 | YES | YES | YES | 7-14 days | 13-36hrs | YES |
|  | Garmin | Fenix series 5/6 x/s | YES | YES | YES | 12 days | 18hrs | YES |
|  | Suunto | Suunto 7 | YES | YES (Google Fit) | YES | Up to 40 days | Up to 12hrs | YES |
|  | Suunto | Spartan Sport | YES | YES (via app) | YES | 7 days | 10hrs | YES |
|  | Suunto | Suunto 9 | YES | YES (via app) | YES | 7 days | 25hrs | YES |
|  | Suunto | Suunto 3 Fitness | YES | YES (via app) | YES | 5 days | 30hrs | YES |
|  | Suunto | Suunto 5 | YES | YES (via app) | YES | 7 days | 20hrs | YES |
|  | Fitbit | Versa 2 | Yes | Fitbit App with premium account | Yes | 6 days | Unknown | No - connect to phone |
|  | Fitbit | Versa Lite | Yes | Fitbit App with premium account | Yes | 4 days | Unknown | No - connect to phone |
|  | Fitbit | Iconic | Yes | Fitbit App with premium account | Yes | 5 days | 10 hours GPS | Yes |
|  | Fitbit | Charge 4 | Yes | Fitbit App with premium account | Yes | 7 days | 5 hours GPS | Yes |
|  | Whoop | 3.0 | Yes | No | No | 5 days | 5 days | No - connect to phone |
|  | MyZone | MZ-60 | Chest strap | Yes | Yes | Replaceable battery - months | Replaceable battery - months | No - connect to phone |
|  | Samsung | Galaxy Watch Active / Active 2 | Yes | Yes but no HR in GPX file | Download app | 7 days | Unknown | Yes |
